# Expression of plasma levels of MMP-2, MMP-9, TIMP-1, and TIMP-2 in patients with abdominal aortic aneurysms

**DOI:** 10.1590/1677-5449.202401631

**Published:** 2025-05-02

**Authors:** Túlio Fabiano de Oliveira Leite, Elpidio Ribeiro da Silva, Karoline Gomes, Daniela Pretti da Cunha Tirapelli, Edwaldo Edner Joviliano

**Affiliations:** 1 Universidade de São Paulo – USP, Faculdade de Medicina de Ribeirão Preto – FMRP, Ribeirão Preto, SP, Brasil.

**Keywords:** abdominal aortic aneurysm, endovascular aneurysm repair, matrix metalloproteinase, tissue inhibitors of metaloproteinases, endoleaks, aneurisma da aorta abdominal, reparo endovascular de aneurisma, metaloproteinase da matriz, inibidores teciduais de metaloproteinases, vazamentos internos

## Abstract

**Background:**

Endovascular aneurysm repair (EVAR) is now considered the preferred treatment modality in most abdominal aortic aneurysm (AAA) patients.

**Objectives:**

The objective of this study was to quantify and evaluate MMP-2, MMP-9, TIMP-1, and TIMP-2 expression response to EVAR based on serum assays at 6-month follow-up.

**Methods:**

47 patients with AAA who underwent EVAR and ten people with no comorbidities were recruited for the study. Plasma levels of MMPs and TIMPs were assayed by ELISA preoperatively and after 6 months in the group submitted to EVAR and only once in the control group. Demographic profiles, clinical follow-up data, and imaging exams with angiotomography performed preoperatively and after 6 months were collected.

**Results:**

Forty-seven patients with AAA were treated with EVAR. 87.2% of these patients were male and 68.08% were smokers. There were no deaths in the first 30 days. Ten patients (21.27%) had an endoleak during the 6-month postoperative period. Higher MMP and TIMP levels were observed in the AAA patients compared with patients in the control group, although without statistical significance. After EVAR, there were increases in MMP and TIMP levels both in the group with endoleaks and in the group without endoleaks (p<0.05). The variables related to demographic and anatomical data and types of devices used by the patients did not show statistical significance, except for a significant reduction in aneurysmal sac diameter (p<0.05).

**Conclusions:**

None of the markers assessed showed any association with endoleak status. However, the concentrations of MMPs and TIMPs in circulation increased in all patients after EVAR. Collectively, these findings suggest that the markers assessed have little potential to influence current post-EVAR monitoring practices.

## Resumo

**Contexto:**

A correção endovascular de aneurisma da aorta (EVAR) é a modalidade de tratamento preferencial para a maioria dos pacientes com aneurisma da aorta abdominal (AAA). O objetivo deste estudo foi quantificar e avaliar os níveis séricos de MMP-2, MMP-9, TIMP-1 e TIMP-2 6 meses após o procedimento.

**Objetivos:**

O objetivo deste estudo foi quantificar e avaliar a resposta da expressão de MMP-2, MMP-9, TIMP-1 e TIMP-2 ao EVAR com base em ensaios séricos no acompanhamento de 6 meses.

**Métodos:**

Foram incluídos 47 pacientes com AAA indicados para EVAR; apenas 10 não tinham comorbidades. A determinação plasmática de MMP-2, MMP-9, TIMP-1 e TIMP-2 foi realizada por ELISA no pré-operatório e aos 6 meses no grupo EVAR e apenas uma vez no grupo controle. Foram coletados dados demográficos, clínicos e exames de angiotomografia realizados no pré-operatório e após 6 meses.

**Resultados:**

Dos 47 pacientes tratados com EVAR, 87,2% eram homens e 68,1%, fumantes. Não houve óbitos em 30 dias, e 10 pacientes (21,27%) apresentaram *endoleak* no pós-operatório. Embora os níveis de MMP-9, MMP-2, TIMP-1 e TIMP-2 tenham sido mais elevados em pacientes com AAA do que no grupo controle, essa diferença não alcançou significância estatística. Observou-se, porém, um aumento significativo desses biomarcadores tanto no grupo com quanto sem *endoleak* após EVAR (p<0,05). As variáveis ​​estudadas relacionadas aos dados demográficos e anatômicos e tipo de dispositivo não apresentaram significância estatística, exceto por uma redução significativa no diâmetro do saco aneurismático (p<0,05).

**Conclusões:**

Nenhum dos marcadores avaliados mostrou associação com a ocorrência de *endoleak*. No entanto, as concentrações circulantes de MMPs e TIMPs aumentaram em todos os pacientes após EVAR. Coletivamente, esses achados sugerem que os marcadores avaliados têm pouco potencial para influenciar as práticas atuais de monitoramento pós-EVAR.

## INTRODUCTION

Formation of abdominal aortic aneurysms (AAA) involves degenerative processes such as upregulation of proteolytic pathways, inflammation, and loss of the arterial wall matrix.^1-4^ Estimated prevalence rates are in the range of 1.3% to 8.9% in men and 1% to 2% in women.^5^ Risk factors for AAA include advanced age, smoking, male sex, pre-existing atherosclerotic disease, dyslipidemia, hypertension, and family history.^6^ Most diagnoses are incidental, and rates have been increasing due to efforts to screen the population at risk.^2,7,8^ Because most aneurysms are asymptomatic, rupture is the most feared complication, with a mortality rate of 80%.^9^

The only definitive treatment for aneurysms is surgery and the endovascular technique is the least invasive.^10^ The most feared complication after endovascular repair is rupture of the aneurysm caused by endoleaks.^11^ Currently, no conservative pharmacological treatment is effective for limiting AAA progression or rupture because of the multiplicity of mechanisms of action involved in AAA development.

Since patients must be monitored after undergoing endovascular repair, there is a need to develop noninvasive methods to identify endoleaks and indicate early intervention. Currently, surveillance tests are dependent on diagnostic imaging methods such as computed tomography (CT) and magnetic resonance imaging (MRI). However, on an experimental basis, biological markers may be promising and can avoid this bias, with the significant advantage of being a minimally invasive option with potentially satisfactory results.^12-15^ Collagen and elastin play important roles in the structure and function of the aorta. Histological studies demonstrate significant changes to the architecture of the aortic wall in aneurysms, with disruption and fragmentation of collagen and elastin fibers and disordered collagen deposition.^16^ These aneurysmal tissue changes are characterized by increased elastolytic and collagenolytic activity caused by increased production of matrix metalloproteinase (MMP) enzymes when compared to normal aortic tissue. The resulting progressive degradation of extracellular matrix proteins is part of the AAA formation process. Their activity in the tissues is regulated by specific tissue inhibitors of metalloproteinases (TIMPs).^17^

The main MMPs involved in aortic aneurysms are gelatinase A (MMP-2) and gelatinase B (MMP-9) and both are capable of degrading the extracellular matrix because they elevate elastolytic and collagenolytic activity when compared with normal aortic tissue. MMP 2 is produced by cells with mesenchymal lineage, whereas MMP 9 is secreted by neutrophils, macrophages, and macrophage-derived osteoclasts. The enzymes responsible for degradation of aortic collagen and elastin, the metalloproteinases, are soluble proteins that are continuously released into the systemic circulation and can be measurable in the plasma of patients with AAA. Elevation of the levels in circulation may serve as a marker for the disease.^18^

The hypotheses to be tested in this study by analyzing plasma levels of MMP-2, MMP-9, TIMP-1, and TIMP-2 in patients affected by AAA and treated by EVAR are as follows: First, plasma MMP-9 and MMP 2 levels will be elevated in AAA-affected patients compared to healthy control subjects. Second, plasma MMP-9 and MMP-2 levels will be lower after successful EVAR treatment. Third, MMP levels will increase during follow-up in patients with endoleaks and consequently continued sac pressurization and possible aneurysm expansion. Fourth, decreased MMP levels during follow-up would indicate effective exclusion of the aneurysm by endovascular treatment.

## METHODS

This prospective study was performed at a center in the Department of Surgery and Anatomy of the Division of Vascular and Endovascular Surgery of the Medical School of Ribeirão Preto at the University of São Paulo between May 2018 and February 2020. Inclusion criteria were patients with infrarenal AAA with or without iliac aneurysms eligible for standard endovascular repair according to current treatment indications. The exclusion criteria were patients with a ruptured aneurysm or aneurysms in any arterial territory other than the infrarenal aorta, patients who (for any reason) did not complete the total follow-up time of 6 months, patients with other underlying diseases (medical psychosis, meningitis, stroke, multiple sclerosis, tumors, and autoimmune diseases), and patients who refused to participate in the study. Ten patients (regardless of ethnicity, age, or sex) without AAA were selected for the patient control group. This project was approved by the Ethics and Research Committee at the Medical School of Ribeirão Preto at the University of São Paulo (Process number: 82522018.0.0000.5440) under registration number 2.525.236.

### Laboratory tests

Peripheral venous blood samples were obtained 24 h before endoprosthesis implantation and 6 months after the procedure. Whole blood was stored in tubes containing ethylenediaminetetraacetic acid (EDTA) and frozen at -80 °C. All materials collected were stored until analysis.

### Metalloproteinases and metalloproteinase inhibitors

Blood samples were collected in tubes containing EDTA anticoagulant, centrifuged at 10,000 g for 10 minutes at 4°C, and then the supernatant obtained was collected and stored at -80°C for testing. In the assays, plasma samples were diluted 50 times for MMP-2 determination, 1000 to 2000 times for MMP-9, and 200 times for TIMP-1 and TIMP-2.

### Determination of MMP-2, MMP-9, TIMP-1, and TIMP-2

Plasma determination of MMP-2, MMP-9, TIMP-1, and TIMP-2 was performed by enzyme-linked immunosorbent assay (ELISA), in accordance with the quantification kit instructions (DuoSet ELISA - R&D Systems). The final step of the assay was performed in a VERSAmax microplate reader (Molecular Devices). The optical density values were read at 450 nm and calculated from a four-parameter standard curve (4-PL) with the aid of SoftMax Pro v5 software. The results obtained were expressed in ng/ml for MMP-2 and in pg/ml for MMP-9, TIMP-1, and TIMP-2.

### Description of devices

Eight different brands of endoprosthesis were used, as follows: the AFX^TM^ (Endologix Inc, Irvine, 146 Calif) (n=4) is an endoprosthesis that features a metallic skeleton of cobalt-chromium coated with e-PTFE and anatomical fixation (fixation of the bifurcation of the endoprosthesis in the bifurcation of the aorta); the GORE^®^ EXCLUDER^®^ (Excluder; W. L. Gore and Assoc, Flagstaff, 147 Ariz) (n=2) features a metal skeleton of nitinol coated with e-PTFE and is a bimodular endoprosthesis with passive proximal fixation (without splinters or hooks for direct fixation to the aortic wall); the MEDTRONIC^®^ ENDURANT® (Endurant; Medtronic, Minneapolis, USA) (n=18) has a metallic skeleton of nitinol coated with polyester and is a bimodular endoprosthesis with active proximal fixation (with barbs for direct fixation to the aortic wall); the APOLLO^®^ (Nano Endoluminal, Florianópolis, Brazil) (n=2) has a nickel-titanium metallic skeleton coated with e-PTFE and is a bimodular endoprosthesis with active proximal fixation; the COOK^®^ ZENITH^®^ (Zenith; CookInc, Bloomington, Ind) (n=5) features a stainless steel skeleton coated with polyester and is a trimodular endoprosthesis with active proximal fixation; the ANACONDA^TM^ (Anaconda; Sulzer Vascutek, Bad Soden, Germany) (n=8) features a nitinol skeleton coated with polyester, is repositionable, and is trimodular with active proximal fixation; the BRAILE^®^ (Braile Biomédica, São José do Rio Preto, Brazil) (n=2) has a metallic nitinol skeleton coated with polyester and is a bimodular endoprostheses with active proximal fixation (without splinters for direct fixation to the aortic wall); the INCRAFT^®^ (n=5) has a metallic nitinol skeleton coated with polyester and is a bimodular endoprosthesis with active proximal fixation (with barbs for direct fixation to the aorta wall); and the Aortic^®^ (Inside Medical Industry, Santa Catarina, Brazil) (n=1) has a nickel-titanium metallic skeleton coated with e-PTFE and is a bimodular endoprostheses with active proximal fixation. The variety of different endoprostheses used was because of the variability of the anatomy of each aneurysm and the most appropriate endoprosthesis was selected for each case.

### Procedures

The procedures were performed under general anesthesia by the same team for all patients, following the institution’s protocols for vascular interventions. Procedures were performed in a surgical center with a GE/OEC 9900 ELITE (GE Hualun Medical Systems Co., Beijing, China) C arm, using bilateral femoral access for delivery and release of the devices. Unfractionated heparin (5,000 units) was administered intraoperatively shortly after insertion of the introducer. A low-osmolarity non-ionic contrast agent (Iopamiron 300; Bracco Imaging SpA, Ferentino, Italy) was used for angiography.

### Follow-up

All patients were followed up at the same outpatient clinic, with return appointments scheduled for 1, 6, and 12 months (the patients were free to visit the outpatient clinic at other times if necessary). At the return visits, patients underwent a thorough clinical examination performed by a trained vascular surgeon. These patients underwent surveillance (total aortic angiography using CT scanning or MRI) at three time points: 1, 6, and 12 months after the surgical procedure, for the purpose of investigating leaks and/or changes in aneurysmal sac morphometry.

### Statistical analysis

Categorical variables were presented as absolute and relative frequencies. Associations between categorical variables were assessed using Fisher’s exact test or the likelihood ratio test.

The Kolmogorov-Smirnov test was used to assess whether quantitative variables were normally distributed. Variables are presented as medians (50th percentile) and interquartile ranges (25th and 75th percentiles). Variables were compared between groups using the Mann-Whitney U test and between time points (preoperative × 1 month, preoperative × 6 months, and 1 month × 6 months) using the Wilcoxon signed-rank test. These results are presented with schematic graphics (boxplots). Statistical significance was set at P <0.05. IBM SPSS Statistics for Windows (version 18.0; Armonk, NY, USA) was used for the calculations and graphics.

## RESULTS

Forty-seven patients with AAA were treated with endovascular techniques. Most patients were male (87.2%) and smokers (68.08%). It was not necessary to exclude any of these patients and there was no need for transfusions in any of the cases. There were no 30-day in-hospital or aneurysm-related deaths. Ten patients (21.27%) had an endoleak during the 6-month postoperative period, nine of whom were male. The types of endoleaks identified were IA, IB, IIA, and IIB in one, one, five, and three patients, respectively, as shown in Table 1.

**Table 1:** Demographic, clinical, and laboratory data for groups with and without endoleaks.

Variable	Patients with endoleaks	Patients without endoleaks	**P**
	n=10	n=37	
Age (years), median (IQR)	72 (67 - 79)	70 (66 - 78)	0.754c
Weight (kg), median (IQR)	76 (65 - 92)	75 (67 - 88)	0.677^c^
Height (m), median (IQR)	1.72 (1.69 – 1.76)	1.67 (1.64 – 1.73)	0.118^c^
BMI (kg/m^2^), median (IQR)	26 (23 - 30)	27 (24 - 30)	0.856^c^
Sex (male)	9 (90%)	32 (86.5%)	1.000^a^
Ethnicity			0.280^b^
White	10 (100%)	32 (86.5%)	
Brown	0 (0%)	2 (5.4%)	
Black	0 (0%)	3 (8.1%)	
Smoking (cigarettes/day), median (IQR)	20 (5 - 40)	20 (10 - 20)	0.663^c^
Neck angulation			0.630^a^
<60°	8 (80%)	32 (86.5%)	
>60°	2 (20%)	5 (13.5%)	
Types of endograft			0.262^b^
AFX	0 (0%)	4 (10.8%)	
Anaconda	2 (20%)	6 (16.2%)	
Aortic	1 (10%)	0 (0%)	
Apollo	1 (10%)	1 (2.7%)	
Braile	1 (10%)	1 (2.7%)	
Cook	0 (0%)	5 (13.5%)	
Gore	0 (0%)	2 (5.4%)	
Incraft	1 (10%)	4 (10.8%)	
Medtronic	4 (40%)	14 (37.8%)	
Type of endograft coating material			0.664^a^
e-PTFE	1 (11,1%)	8 (21.6%)	
Polyester	8 (88.9%)	29 (78.4%)	
Contrast volume (mL)*	145 (115 - 200)	120 (100 - 150)	0.233^c^
Radiation (scopy time/sec)*	1702 (1409 - 2030)	1569 (1261 - 2194)	0.649^c^
Radiation (cumulative dose/mgy)*	368 (244 - 445)	337 (232 - 429)	0.687^c^
Surgery time (min)*	120 (110 - 148)	123 (106 - 173)	0.696^c^
Diameter (cm) AAA	6 (6 - 7)	6 (6 - 7)	0.297^c^
			0.047^c^
Proximal neck diameter (cm)*	2.55 (2.33 – 2.73)	2.30 (2.10 – 2.50)	
Maximum diameter right distal neck (cm)*	1.45 (1.15 - 1.53)	1.30 (1.13 - 1.50)	0.462^c^
Maximum diameter left distal neck (cm)*	1.20 (1.20 - 1.43)	1.30 (1.00 - 1.50)	0.844^c^
Proximal neck length (cm)*	2.65 (2.08 - 3)	2.90 (2.03 - 3.90)	0.593^c^
Thrombus diameter in AAA*	0 (0 - 22)	0 (0 - 30)	0.298^c^
Oversize proximal neck (%)*	20 (14 - 20)	20 (15 - 20)	0.829^c^
Oversize right distal neck (%)*	18 (10 - 20)	20 (20 - 20)	0.044^c^
Oversize left distal neck (%)*	18 (10 - 20)	20 (20 - 20)	0.044^c^
Number of lumbar arteries*	4 (3 - 5)	4 (2 - 5)	0.635^c^
Diameter of lumbar artery (cm)*	0.25 (0.20 - 0.30)	0.20 (0.20 - 0.30)	0.853^c^
IMA diameter (cm)*	0.3 (0 - 0.3)	0.2 (0 - 0.3)	0.540^c^
Thrombosis (%)			0.875^b^
0	7 (70%)	28 (77.8%)	
25-50	2 (20%)	5 (13.9%)	
>50	1 (10%)	3 (8.3%)	
Blood Loss (mL)*	270 (190 - 313)	290 (200 - 358)	0.686^c^
Endoleak (IPO)	9 (90%)	3 (8.1%)	<0.001^a^
Type			<0.001^b^
IA	2 (22.2%)	0 (0%)	
IIA	3 (33.3%)	3 (100%)	
IIB	4 (44.5%)	0 (0%)	
Endoleak (6 months)	9 (90%)	0 (0%)	<0.001^a^
Type			
IA	1 (10.0%)	0 (0%)	
IB	1 (10.0%)	0 (0%)	
IIA	5 (50.0%)	0 (0%)	
IIB	3 (30.0%)	0 (0%)	

IQR: interquartile range. IPO: immediate postoperative period. IMA: inferior mesenteric artery; cm: centimeter.

* Median (interquartile range).

^a^: Fisher’s exact test.

^b^: likelihood ratio test.

^c:^ Mann-Whitney test.

Higher MMP-9, MMP-2, TIMP-1, and TIPM-2 levels were observed in AAA patients compared to the patients in the control group, although without statistical significance, as shown in Table 2. After EVAR, there were increases in MMP-9, MMP-2, TIMP-1, and TIMP-2 both in the group with endoleaks and in the group without endoleaks (p<0.05), as shown in Table 3

**Table 2:** Laboratory data for control and study groups.

Variable	Control	Study (preoperative)	Study (6 months)	P_1_	P_2_	P_3_
Median (**IQR**)	(n=10)	(n=47)	(n=47)			
MMP-2**(ng/ml)**	374 (331 - 411)	366 (323 - 433)	436 (393 - 504)	0.867	0.003	0.003
MMP-9(pg/ml)	1806842 (1519842 - 2576244)	2764326 (1650344 - 4306912)	3320696 (1493512 – 5098956)	0.102	0.126	0.126
TIMP-1 (pg/ml)	132357 (99515 - 152834)	150318 (134823 - 197189)	189566 (139233 - 233892)	0.054	0.014	0.014
TIMP-2 (pg/ml)	92693 (83047 - 107185)	102676 (89406 - 117338)	117375 (102095 - 133995)	0.224	0.002	0.002
TIMP-1 (pg/ml)	132357 (99515 - 152834)	150318 (134823 - 197189)	189566 (139233 - 233892)	0.054	0.014	0.014
TIMP-2 (pg/ml)	92693 (83047 - 107185)	102676 (89406 - 117338)	117375 (102095 - 133995)	0.224	0.002	0.002

P1 [Control x Study comparison (preoperative)]: Mann-Whitney test; P2 [Comparison Control x Study (6 months)]: Mann-Whitney test; P3 [comparison Study (preoperative) x Study (6 months)]: Wilcoxon signed rank test. IQR: interquartile range. ng/ml: nanogram per ml; pg: picogram per ml.

**Table 3:** Laboratory data for groups with and without endoleaks.

Variable	Patients with endoleaks	Patients without endoleaks	P^c^
Median (IQR)	n=10	n=37	
MMP-2 (ng/ml)			
Preoperative	341 (286 - 411)	366 (340 - 433)	0.132
6 months	438 (347 - 505)	434 (396 - 505)	0.435
p (preop. x 6 months)***	0.022	<0.001	
MMP-9 (pg/ml)			
Preoperative	3284647 (1108374 - 4818180)	2749133 (1969572 - 4247284)	0.938
6 months	2826677 (1659062 - 3378612)	3336122 (1474335 - 5276627)	0.483
p (preop. x 6 months)***	0.386	0.464	
TIMP-1 (pg/ml)			
Preoperative	134315 (127503 - 148103)	165198 (139697 - 207404)	0.024
6 months	174615 (123545 - 216289)	191211 (145643 - 236588)	0.323
p (preop. x 6 months) ***	0.028	0.017	
TIMP-2 (pg/ml)			
Preoperative	95518 (81942 - 113607)	103098 (90468 - 118358)	0.232
6 months	115890 (89781 - 129095)	118564 (102457 - 134638)	0.377
p (preop. x 6 months) ***	0.017	<0.001	
Diameter (cm)			
Preoperative	6.2 (5,7 - 7,0)	5.8 (5.5 – 6.6)	0.297
1 month	5.9 (5.4 – 7.1)	5.6 (5.2 – 6.4)	0.226
6 months	5,9 (5,4 - 6,8)	5,3 (4,9 - 6,1)	0.048
p (pre x 1 month) ***	0,127	0,091	
p (pre x 6 month) ***	0,001	<0,001	
p (1 month x 6 month) ***	1,000	0,001	

IQR: interquartile range; cm: centimeter; preop.: preoperative.

*** Wilcoxon signed rank test.

c: Mann-Whitney test.

None of the variables related to demographics, anatomical data, procedure, or type of devices implanted showed any statistical significance, except for a significant reduction in aneurysmal sac diameter (p<0.05).

## DISCUSSION

MMPs are a constantly expanding family of endopeptidases (25 identified so far in vertebrates, 23 of which are found in humans), with proteolytic activity towards one or more components of the extracellular matrix.^19^ The MMPs are connective tissue-degrading enzymes that participate in a variety of physiologic remodeling processes and in many diseases associated with excessive tissue degradation, such as arthritis, tumor invasion, periodontitis, and osteoporosis.^20^ Development of abdominal aortic aneurysms (AAA) is characterized by inflammation, degradation, and remodeling of the aortic wall. Collagen and elastin are the main aortic wall proteins that ensure the structural integrity and mechanical properties of the normal aorta. Rupture, fragmentation of elastin fibers, and disordered collagen deposition are histological changes found in the architecture of the arterial wall in aortic aneurysms.^16^

There are theories that determination of the success of EVAR could be based on the reduction of biomarkers secreted by the AAA.^21^ So far, relatively few studies have specifically investigated the association of blood markers with the presence of endoleak, and the potential value of an approach to EVAR surveillance based on blood markers remains unclear.

Several previous investigations have evaluated the relationship between circulating concentrations of MMPs and TIMPs and the presence of endoleak and treatment markers after EVAR, although the significance and extent of reported associations vary between studies (see Table 4).^14,17,22-27^ A meta-analysis published in 2015 identified a significant positive association between plasma MMP-9 concentration and presence of endoleak.^12^ The findings of that meta-analysis contradict the results of the present prospective study, which analyzed a larger sample than the studies included in the meta-analysis and found no relationship between endoleak diagnosis and circulating concentration of MMPs or TIMPs. Another study, published by Moxon et al.^7^ in 2017, also found no relationship between endoleak diagnosis and MMP-9 and also had a larger sample size than the studies included in the meta-analysis.^2^ The reasons for this discrepancy may be related to differences in the populations studied, different handling of blood samples, lack of standardization between EVAR time and measurement of biomarkers, or lack of standardization of the image surveillance protocol and evaluation methods. One study involved administration of azelnidipine (a calcium channel blocker) which may possibly contribute to a decrease in plasma inflammatory biomarkers.^24^ Furthermore, Monaco et al.^22^, for example, specifically investigated endoleaks after EVAR for descending thoracic aortic aneurysms, rather than AAA as in the present study, and pathophysiological differences may complicate the comparison, in addition to making the study heterogeneous, potentially causing the conflicting result. For greater analytical power, further studies employing large cohorts of patients are needed to more definitively assess the associations between circulating concentrations of MMPs and TIMPs and presence of endoleaks.

**Table 4:** Case series.

Study	Study design	Type of study	Number of EVARs	Endoleak types (n=number)	Aneurysm type	Imaging surveillance protocol	EVAR stent graft type	Biomarkers measured	Time between EVAR and MMP measurement	Association between biomarkers and endoleaks after EVAR	Method of quantitative biomarkers assessment
Monaco et al., 2007^22^	Evaluation of plasma MMP-9, MMP-3 and TIMP-1 levels in patients undergoing EVAR vs. healthy volunteers (20 vs 25)	Prospective cohort	20	Type I:2	TAA	CTA on discharge and 1 and 6 months postoperatively	Talent (Medtronic)	MMP-9	Baseline	Positive	ELISA
				Type II:2				MMP-3	1 month		
				-4				TIMP-1	3 months		
									6 months		
Hellenthal et al., 2012^14^	Evaluation of plasma MMP-9 levels in patients undergoing EVAR	Prospective cohort	37	Type I:4	AAA	CTA (period not stated)	NR	MMP-9	Baseline	Positive	ELISA
				Type II:12				MMP-2	18 months		
				Type III:1				TIMP-1	21 months		
				-17							
Sangiorgi et al., 2001^17^	Evaluation of plasma MMP-9 and MMP-3 levels in patients undergoing open surgery or EVAR (15 vs 30)	Prospective cohort	30	Type I:5	AAA	CTA, 6 months	Gore Excluder (n =16)	MMP-9	Baseline	Positive	ELISA
				Type II:2		postoperatively and on onset of new symptoms	Vanguard (n= 7)	MMP-3	1 month		
				-7			Talent World Medical (n= 7)		3 months		
									6 months		
Lorelli et al., 2002^23^	Evaluation of plasma MMP-9 levels in patients undergoing open surgery or EVAR (26 vs. 18)	Prospective cohort	18	8 (endoleak types not reported)	AAA	CTA, 1 and 3 months postoperatively. CTA, before discharge and at 3 months postoperatively (custom-made stent graft)	AneuRx Medtronic (n =6)	MMP-9	Baseline	Positive	ELISA
							Ancure Guidant (n =16)		1 month		
							Custom made Malmo-Ivancev (n =3)		3 months		
Nakamura et al., 2009^24^	Evaluation of plasma MMP-9 in patients undergoing EVAR randomized to receive azelnidipine and control (12 v 10)	Prospective Cohort	22	4 (endoleak types not reported)	AAA	CTA (preoperatively and 3 months and 1 year postoperatively)	Cook Zenith (7 in azelnidipine, 6 in control)	MMP-9	Baseline	Positive	ELISA
							Gore Excluder (5 in azelnidipine, 4 in control)		1 month		
									3 months		
Marchetti et al., 2017^25^	Evaluation of plasma MMP-9 levels in patients undergoing open surgery or EVAR	Prospective cohort	22	5 (endoleak types not reported)	AAA	CT after 1, 3 and 6 months, and then annually. postoperatively	NR	MMP-9	Baseline	Positive	ELISA
									1 month		
									3 months		
									6 months		
Taurino et al., 2004^26^	Evaluation of plasma MMP-9 levels in patients undergoing open surgery or EVAR	Prospective cohort	9	NR	AAA	CT and ultrasound examination after 1 month	NR	MMP-9	Baseline	Positive	ELISA
									1 week		
									1 month		
Moxon et al., 2017^27^	Evaluation of plasma MMP-9, OPG, D-dimer, HCY, and CRP levels in patients undergoing EVAR	Prospective cohort	75	Type I: 2	AAA	CT after 1, 6, 12, 24, and 36 months.	NR	MMMP-9	Baseline 3 months	Negative	NR
				Type II: 20				OPG			
				Type I and III: 1				D-dimer			
				One endoleak could not be classified (24)				HCY			
								CRP			
Current Study	Evaluation of plasma MMp-2, MMP-9, TIMP-1 and TIMP-2 levels in patients undergoing EVAR	Prospective cohort	47	Type IA:1	AAA	CT after 1, 6, and 12 months.	AFX (n=4)	MMP-9	Baseline 6 months	Negative	ELISA
				Type IB:1			Gore Excluder (n=2)	MMP-2			
				Type IIA:5			Medtronic Endurant (=18)	TIMP-1			
				Type IIB:3			Apollo (n=2)	TIMP-2			
							Cook Zenith (n=5)				
							Anaconda (n=8)				
							Braile (n=2)				
							Incraft (n=5)				
							Aortic Inside (n=1)				

EVAR: endovascular aneurysm repair. AAA: abdominal aortic aneurysm. CTA: computed tomography angiography. MMP-2: matrix metalloproteinase-2. MMP-3: matrix metalloproteinase-3. MMP-9: matrix metalloproteinase-9. TAA: thoracic abdominal aneurysm. TIMP-1 tissue inhibitor of metalloproteinase-1. TIMP-2 tissue inhibitor of metalloproteinase-2. NR: not reported.

The current main treatment for AAA is endovascular aneurysm repair (EVAR). Despite the low perioperative morbidity and mortality, the durability of EVAR is a matter of concern, as some patients may experience AAA sac leaks or perfusion. Patients undergoing EVAR require long-term monitoring with imaging tests such as angiotomography, magnetic resonance angiography, or Doppler ultrasound to detect endoleaks. These have disadvantages such as repeated exposure of the patient to ionizing radiation, use of contrast in patients with impaired renal function, and the need for specialized infrastructure, generating a negative impact on the cost-effectiveness of EVAR.^27^

An ideal biomarker for use in endoleak detection would have high sensitivity to detect all endoleaks, as well as high specificity to exclude individuals without endoleaks and the ability to differentiate each type of endoleak. Only one study carried out an analysis with sensitivity and specificity, but it was not able to correlate with the types of endoleak, which is extremely important since type I and type III endoleaks have a high potential for rupture when not corrected.^14^ In addition, measuring biomarkers is practically unfeasible outside the research environment, because the tests are expensive and require technology that not all laboratories are able to acquire, in addition to a lack of standardization of techniques.

There are several conflicting factors that can alter the plasma levels of biomarkers, such as autoimmune diseases, neoplasms, medications, and atherosclerosis. So far, no studies have been able to differentiate the plasma levels of these biomarkers between AAA and atherosclerosis, which is commonly present in patients with AAA. Besides, it is important to remember that after endovascular treatment of AAA, the aneurysm wall is still present, as this technique only excludes the aneurysmal sac. This may be a confounder of MMP and TIMP concentrations post-EVAR. These biomarkers are also unlikely to be able to determine the type of endoleak after EVAR, as the characteristics of the population and the anatomy of the aneurysms are very heterogeneous. Perhaps one line of research could be to develop medications that could act on MMPs and TIMPs in order to control the progression of the disease as a targeted therapy for atherosclerosis and AAA, without the need for EVAR.

Several limitations of this study merit consideration. The small number of patients overall as well as a low number of patients with an endoleak. The patient sample is small, which means there may be confounding factors such as age, gender, or lifestyle factors (e.g., smoking) that could influence MMP expression. The use of a previously described MMP without performing a study of paired controls may not be reproducible, since the MMPs involved in the pathophysiology of AAA are not very well known. Although the clinical potential of MMPs as biomarkers is promising, there are still challenges that need to be overcome before practical implementation, such as standardization of the method, requiring development of uniform protocols for collection, processing and analysis, clinical validation with large-scale studies, integration with clinical practice, and accessibility for all patients with tools for routine application.

## CONCLUSION

This study evaluated the potential for use of circulating concentrations of MMP-2, MMP-9, TIMP-1, and TIMP-2 as diagnostic markers for endoleak or treatment of AAA by EVAR. None of the markers assessed showed any association with endoleak status. However, circulating concentrations of MMPs and TIMPs increased in all patients after EVAR. Collectively, these findings suggest that the markers evaluated have little potential to influence current post-EVAR monitoring practices.
